# The impact of RAS on cell differentiation in health and disease

**DOI:** 10.1042/BCJ20253364

**Published:** 2025-11-17

**Authors:** Carla Jane Duval, Sara Bottone, Elisabeth Schaffner-Reckinger, Daniel Kwaku Abankwa

**Affiliations:** 1 Cancer Cell Biology and Drug Discovery Group, Department of Life Sciences and Medicine, University of Luxembourg, Esch-sur-Alzette, 4362, Luxembourg

**Keywords:** cancer, differentiation, developmental biology, MAPK, RASopathy, stem cell

## Abstract

RAS proteins have been studied for more than 40 years, mainly in the context of cancer. Given that RAS signaling promotes cell cycle progression, it is commonly assumed that its main function is to drive cell proliferation. In this review, we will, however, address the roles of RAS during cell differentiation, which is intertwined with cell division during organismal development and tissue homeostasis in the adult. Our analysis suggests a far-reaching and profound impact of RAS signaling in associated processes. During vertebrate embryonal development, the FGF-RAS-ERK signaling axis is involved as early as germ layer induction and embryonal patterning. Current evidence suggests that RAS fundamentally controls the balance between stem cells and their differentiated progeny. RAS signaling needs to be downmodulated to sustain pluripotent stem cells. Inhibition of RAS activity is also required to preserve adult stem cell quiescence. At the other end of the differentiation spectrum, a different kind of RAS inactivation by the GTPase-activating protein (GAP) neurofibromin 1 (NF1) appears central to permit terminal differentiation, e.g., of muscle tissue. This latter process is disabled in muscle-borne cancer and likely also in other cancer types. In the RAS-associated developmental diseases, the RASopathies, cell differentiation appears to be broadly perturbed throughout development. We suggest that loss of RAS pathway activity mainly manifests in the stem/progenitor cell compartment, whereas inhibition of NF1 GAP-mediated RAS inactivation blocks terminal differentiation. Given that defects accumulate during development, it is plausible to assume that only progressive pathological phenotypes of RASopathies offer a realistic chance for treatment, notably by repurposing RAS-MAPK pathway oncology drugs. Thus, the impact of RAS on cell differentiation represents, in our view, the common mechanistic denominator of cancer and RASopathies. We conclude by giving a perspective on how improving our insight into the functioning of RAS during cell differentiation could lead to the development of misdifferentiation-correcting drugs.

## Introduction

The human genome encodes 40 different RAS proteins [1], of which only four HRAS, NRAS, and the splice variants KRAS4A and KRAS4B, are intensively studied. This is because the genes of these proteins are collectively mutated in ~19% of cancer patients and thus amongst the most frequently mutated oncogenes [2].

**Figure 1:**
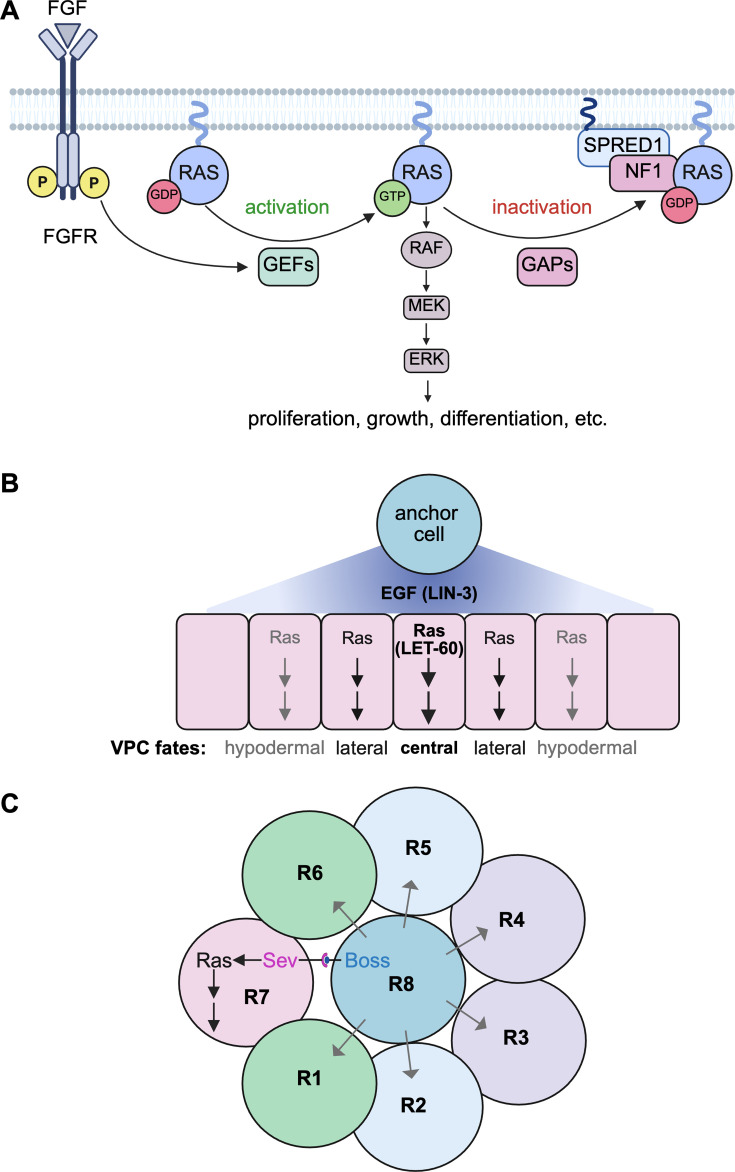
Classical examples of the RAS-MAPK pathway guiding cell differentiation in model organisms. (**A**) The classical topology of the RAS-MAPK pathway was established in genetic experiments and biochemical interaction studies. The pathway is highly conserved throughout evolution and widely employed during organismal development. (**B**) Example of an EGF gradient (i.e. a morphogen gradient) driven RAS-MAPK-dependent gene expression program, which is only activated above a threshold leading to cells adopting distinct vulval fates. In *C. elegans*, the patterning of vulval precursor cell (VPC) fates is to a significant extent directed by EGF (LIN-3) signaling through the RAS-MAPK pathway, where the single RAS ortholog LET-60 is essential for inducing vulval development [3]. During larval development, the so-called anchor cell releases EGF, forming a concentration gradient that determines VPC fates. The cell closest to the anchor cell adopts the central vulval fate, while neighboring cells adopt lateral vulval cell fates, and the farthest cells become non-vulval, hypodermal cells due to minimal EGF exposure [4]. (**C**) Example of cell–cell contact and morphogen-mediated cell differentiation. In *D. melanogaster*, the RAS-MAPK pathway is crucial for compound eye development [5]. Within each of the 800 identical compound eye units, the photoreceptor cell R8 is blue/green-sensitive and the adjacent R7 cell is UV-sensitive. The specification of R7 depends on RAS signaling, which is triggered by activation of the receptor tyrosine kinase Sev on the R7 surface by the ligand Boss, expressed on the already differentiated R8 cell. The R8 cell recruits sequentially all surrounding cells and, by secreting the EGFR ligand Spitz (gray arrows), promotes their differentiation into photoreceptor cells.

All RAS proteins belong to the family of small GTPases and function as membrane-anchored molecular switches, which are active when GTP-bound and inactive in the GDP state. Canonical activation is triggered by guanine nucleotide exchange factors (GEFs), such as Son of Sevenless 1, which allow the exchange of GDP for GTP that is present in excess in cells. GEFs are indirectly activated by several plasma membrane receptors, which integrate environmental stimuli such as growth factors (e.g. EGF and FGF), cytokines (e.g. SDF-1/CXCL12), morphogens (e.g. TGFβ) and cell-surface ligands (e.g. extracellular matrix proteins). The conformational change following GTP-binding enables activated RAS to recruit effectors as downstream interaction partners, such as RAF proteins, which trigger the MAPK pathway (Figure 1A). MAPK pathway activity during the G1 cell cycle phase increases the expression of cyclin D, thus driving cell cycle progression and cell proliferation [6]. It is this activity that is commonly associated with RAS driving cell division. Other activities downstream of RAS are the regulation of apoptosis/cell survival, cell metabolism/growth, and cell differentiation [7].

RAS on its own is a poor GTPase; therefore, it requires GTPase-activating proteins (GAPs) for efficient GTP hydrolysis and inactivation. The GAP with the highest disease relevance is neurofibromin 1 (NF1), which is recruited to the plasma membrane by one of three SPRED paralogs [8]. SPRED itself is recruited by RAF proteins to nanodomains of active KRAS4B [9]. In addition, dephosphorylation of Y420 of SPRED1 by the SHP2 phosphatase appears to inhibit SPRED1 activity [10]. Importantly, it is the activity of NF1 and related arginine-finger GAPs that is disabled by oncogenic hotspot mutations in RAS [11,12]. The preeminent importance of NF1-dependent processes in cancer is further supported by the fact that GTP hydrolysis of major oncogenic RAS mutants can in fact be stimulated by the GAP RGS3, which appears to rely on a key asparagine for catalysis [13]. Thus, GEFs and GAPs are part of the basic regulation loop of RAS, which is also reflected in the typical depiction of the RAS activation cycle (Figure 1A).

It is generally accepted that dysregulation of RAS activity drives life-threatening diseases such as cancer and a spectrum of rare diseases called RASopathies [14]. The assumption is that mutationally increased RAS activity overstimulates all downstream processes, such as proliferation, growth, etc. This disease focus has led to studying RAS almost exclusively in aberrant cancer cells, which has helped little to understand how RAS normally functions throughout ontogenesis on the cellular scale.

During organismal development, the RAS pathway (among many other signaling pathways) is recurrently used as cells divide (proliferate) and gradually adopt distinct specializations (differentiation), such as that of a neuron or muscle cell. Proliferation and differentiation are intimately linked during development, where only stem and progenitor cells proliferate, before committing to a specific lineage during differentiation [15]. In the adult, related processes are still needed for tissue homeostasis and repair. A regular turnover takes place, as cells are dying and replaced by the proliferation of stem/progenitor cells residing within different tissues. For example, in the gut, cell division takes place once a day, and the whole tissue is replaced every four days [16]. Recurrent use of signaling pathways is possible as their activity is spatio-temporally regulated not only within one cell but across many cells and tissues that emerge and become increasingly specialized as an organism develops.

Already in the late 1980s and 1990s, classical experiments had solidly associated RAS signaling with cell differentiation, such as during *Caenorhabditis elegans* vulva development (Figure 1B) or *Drosophila melanogaster* compound eye development (Figure 1C). These examples illustrate how the same pathway produces different cell fate outcomes depending on the position of the cell in the developing tissue and the developmental time point, hence the natural parameters that typically determine the signaling environment and the cell state. Furthermore, genetic experiments in these model organisms enabled not only the discovery of RAS pathway components but also established their hierarchical genetic dependence as commonly seen in signaling pathway diagrams [17].

Another variation of this theme is the differential expression of gene paralogs in higher organisms, which often execute the same biochemical function but at a distinct developmental time point or in a specific tissue context [18]. At the same time, partial overlap of paralog activity is important to ascertain the robustness of developmental processes. However, the cancer-associated RAS paralogs (or isoforms, as typically referred to in the community) are ubiquitously expressed [19]. Hence, the question remains in which context and how HRAS, NRAS, KRAS4A, and KRAS4B perform their distinct functions. Sparse evidence suggests they affect different stages of cell differentiation, with KRAS4B promoting stemness on one end of the spectrum and HRAS enhancing cell differentiation on the other end [20,21]. Curiously, this order correlates with that of their exploitation in cancer, where *KRAS* is most and *HRAS* least frequently mutated [2]. Does this mean that the cancer-relevant function of these RAS isoforms is rooted in their role during cell differentiation?

To address this provocative question, we will here review how RAS is involved in regulating cell differentiation from the earliest stages of vertebrate embryonal development during blastocyst formation to the mature organism. Using mostly muscle tissue as an example, we will highlight how the two major disease groups that are associated with aberrant RAS activity, RASopathies and cancer, can be regarded as diseases of aberrant cell differentiation. Given that RAS overactivation is commonly associated with these diseases, we look at how and when during cell differentiation the negative regulators of RAS come into play. We conclude that the role of RAS in cell differentiation should be more intensively studied and implemented, e.g. in future drug development programs.

## Cell differentiation is fundamental for organismal development and repair

The fertilized egg, the zygote, gives rise to anatomically complex organisms after numerous rounds of cell divisions and cell specializations during ontogenesis. At the beginning of embryonal development, three primordial tissues, the germ layers, are specified, from which all major tissues of the body are derived [22]. Cell proliferation and differentiation go hand-in-hand as the organism grows, tissues are patterned, and distinct organ systems are formed.

**Figure 2:**
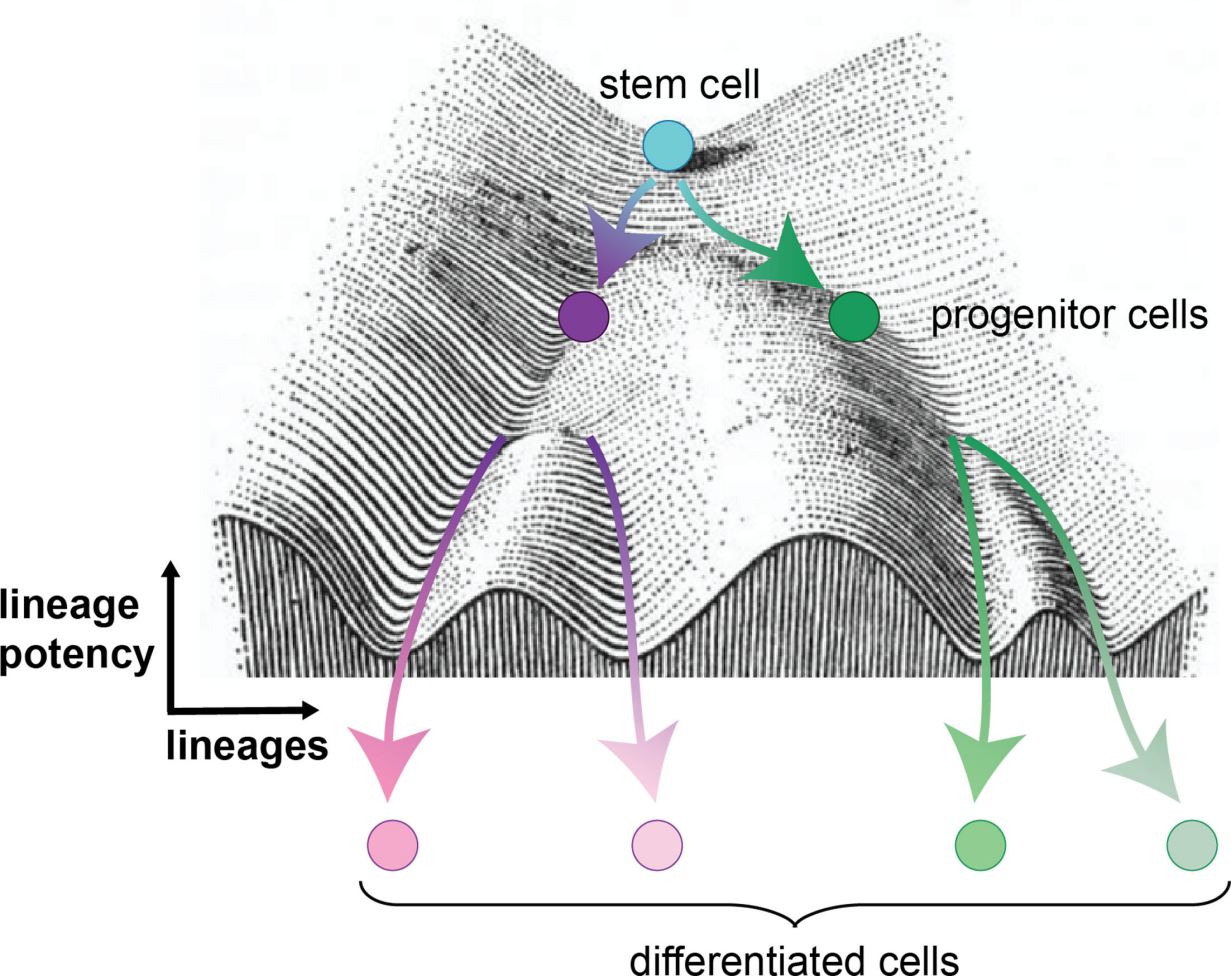
Waddington’s landscape model illustrating cell lineage determination during development. A stem cell transitions from a highly potent state through intermediate states of committed progenitors, which can give rise to one or several further differentiated cell types. Valleys denote specific lineage trajectories, while bifurcations indicate fate decisions.

In this process, stem cells serve as founder cells for a certain developmental or tissue lineage. They are defined by their ability to self-renew, i.e. divide to create further stem cells with equal self-renewal potency. Potency describes the ability of the stem cell to generate several different types of cells. Their progeny are typically progenitors, which possess a more restricted capacity to self-renew and are already partially committed to follow a distinct developmental path, i.e. their developmental fate or lineage is more determined toward a certain tissue type. Thus, on their way to their final differentiated state, cells gradually assume distinct intermediate states, lose potency, and become more lineage-restricted, where the definition of stem and progenitor cells becomes relative (Figure 2).

In the adult organism, so-called adult or somatic stem cells have been found in several tissues (e.g. skin, muscle, gut epithelium, hematopoietic system, liver, pancreas, and brain), where they ascertain tissue homeostasis and enable limited repair of damaged tissue [22,23]. These cells must remain in a reversible quiescent state to preserve the stem cell pool until activated for tissue repair [23]. Activated stem cells can divide symmetrically (two cells with the same potency, i.e., proliferation) or asymmetrically (two cells with different potencies) to restore tissue integrity [15]. During asymmetric divisions, multiple factors enable the two daughter cells to assume distinct cell fates, such as asymmetrically distributed polarity proteins, cellular organelles, and molecular components of the stem cell niche itself [24,25]. It is plausible to assume that this mode of cell division is particularly important for tissue homeostasis in the adult, as it preserves the stem cell pool while generating more differentiated progeny necessary for repair.

**Figure 3:**
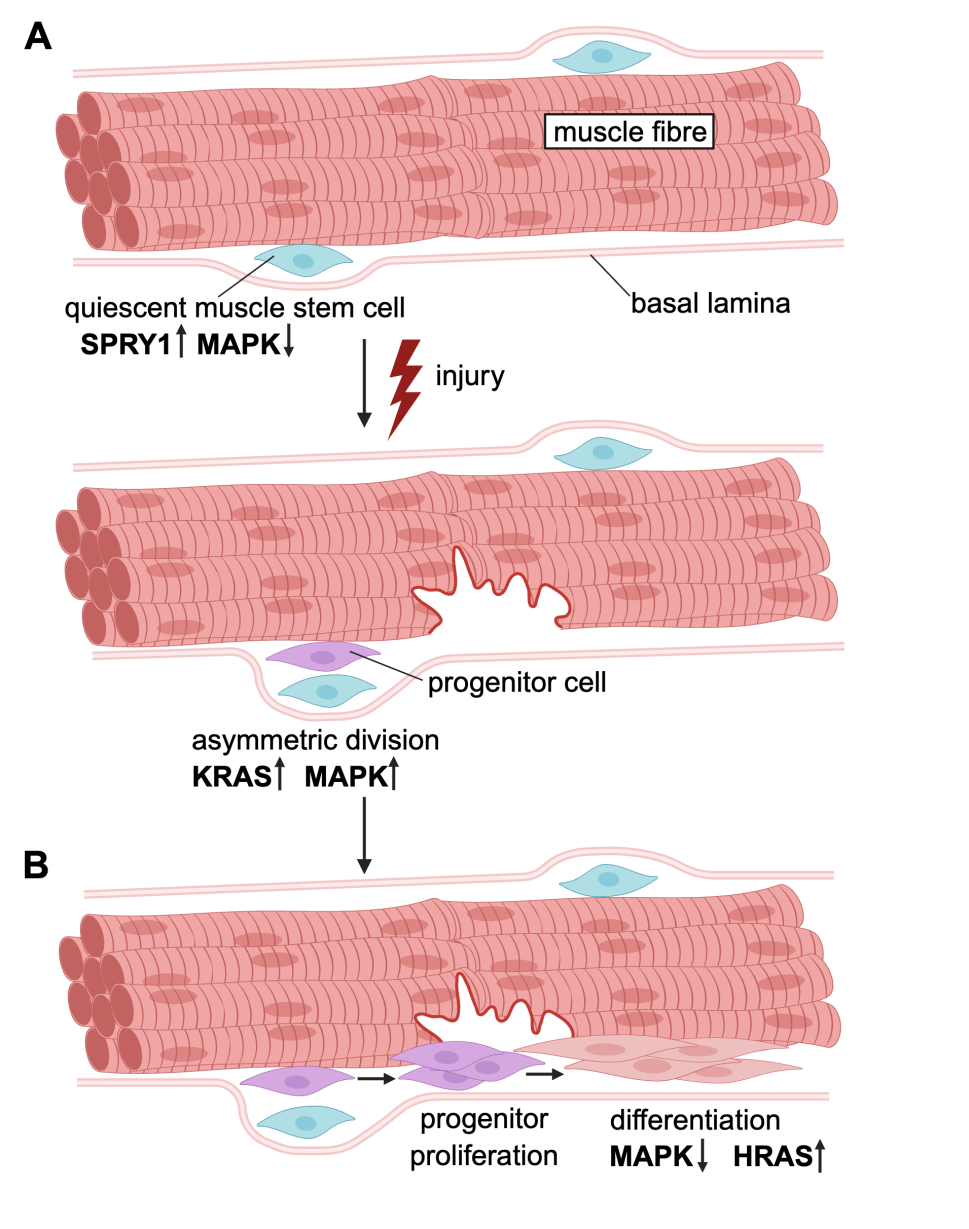
To repair tissue damage in the adult, muscle stem cells divide asymmetrically**,** yielding proliferative progenitors that differentiate into muscle fibers. (**A**) Muscle stem cells reside between the muscle fiber and the basal lamina in a quiescent state that is maintained by high levels of the MAPK-suppressor SPRY1. After an injury, they become activated and undergo apical-basal asymmetric divisions (bottom), which require KRAS-MAPK activity. The daughter cell close to the basal lamina retains the stem cell properties, while the other daughter cell toward the muscle fibre becomes a committed muscle progenitor. (**B**) Progenitors expand proliferatively, and terminal differentiation appears to be licensed by the transient NF1-mediated suppression of MAPK activity. During later stages of differentiation that involve fusion of myocytes into myotubes that become integrated into the muscle fiber for repair, HRAS becomes up-regulated. SPRY, Sprouty.

A well-studied example that illustrates these processes is that of muscle development and repair [26]. During development, specific muscle progenitor cells that are derived from certain early tissues of the primordial body plan become quiescent as muscle-resident stem cells, called satellite cells [27,28]. Upon injury or stimulation, these stem cells divide asymmetrically in a specific orientation, where the committed progenitor cell emerges on the side facing the muscle fiber, while the self-renewed stem cell faces the basal lamina, which surrounds the muscle fiber [29] (Figure 3A). Progenitors then transiently proliferate (hence they are also referred to as transient amplifying cells) to supplement the repair site before starting to differentiate in a complex process from myocytes via myotubes into muscle fibers (Figure 3B). Importantly, during aging, the number of muscle stem cells is progressively lost, thus diminishing the regenerative capacity of muscle [30].

Evidence suggests that other tissues follow a similar hierarchical differentiation trajectory [23]. Most complex in this regard is probably brain development, where differentiation trajectories are highly dependent on intrinsic and extrinsic factors as more than 3000 neuron subtypes are generated [31,32]. We will next explain how RAS signaling is implicated in organismal development and stem cell maintenance.

## The normal function of RAS signaling during development: from stem cells to tissue specialization

RAS is typically associated with cancer, where it is believed to mainly drive excessive proliferation. However, this is not the ‘day job’ of RAS. In this chapter, we will look at the normal functioning of RAS during healthy organismal development.

**Figure 4:**
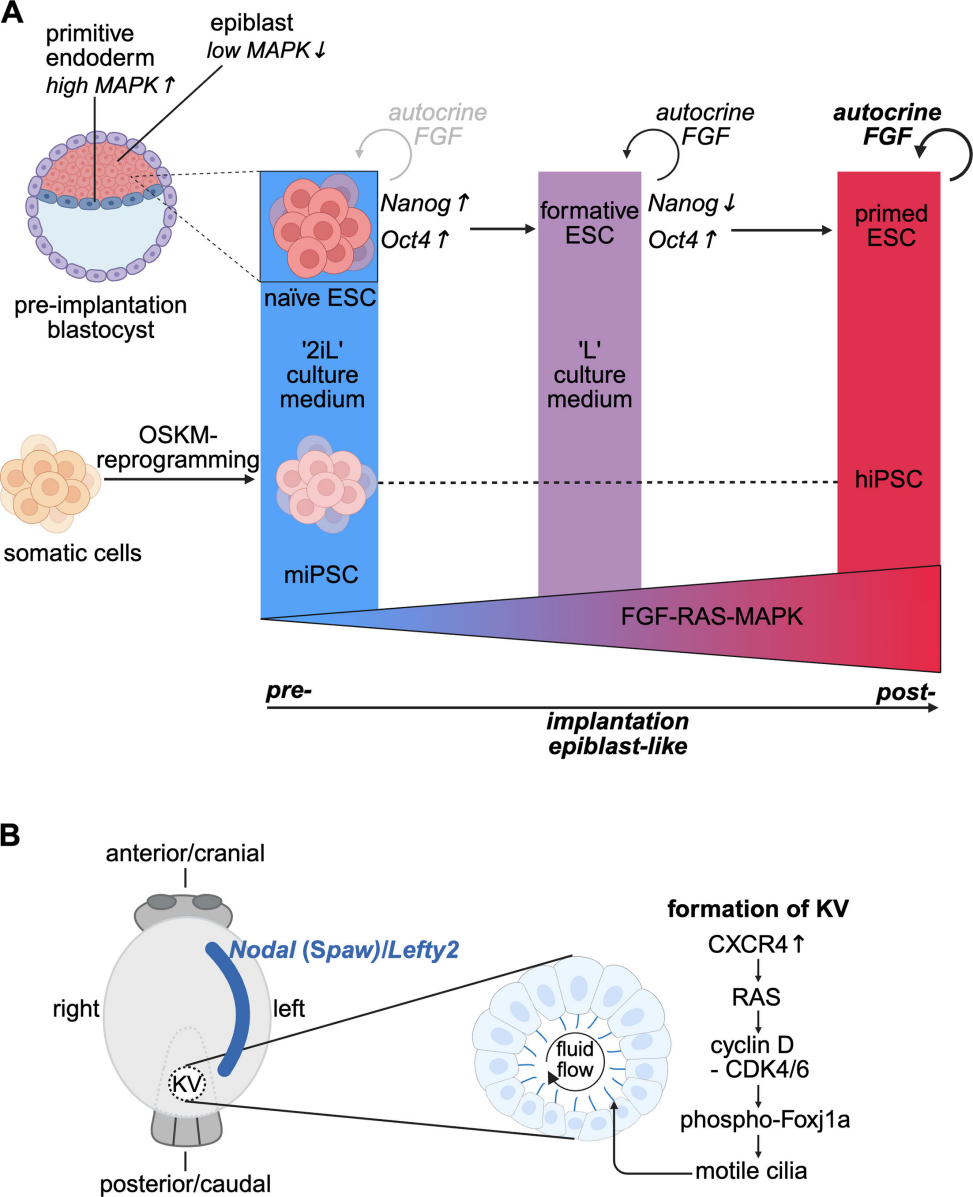
Early cell fate specification and embryonal patterning require RAS signaling**.** (**A**) The first cell fate specifications during the blastocyst stage lead to the emergence of the epiblast and the primitive endoderm, which are derived from the inner cell mass of the blastocyst. Pluripotent naïve mouse ESC are derived from the epiblast and are cultured in ‘2iL’ medium, containing a MAPK- and a Wnt-pathway inhibitor (‘2i’) and the cytokine LIF (‘L’). The derived formative state is stable in ‘L’ medium, and upon 2iL removal, it can transition to the primed ESC state, which is more restricted in its developmental potential. Somatic cells can be reprogrammed into iPSCs, which are supported by 2iL medium. Interestingly, the developmental potential of mouse iPSC (miPSC) appears greater than that of human iPSC (hiPSC). (**B**) Ventral view of the zebrafish embryo and magnification of Kupffer’s vesicle (KV), which is a transient organ present early in development. Each cell of the single-cell layer surrounding the fluid-filled lumen carries cilia, of which 80% are motile. Formation of motile cilia of the KV is promoted by a CXCR4-RAS-dependent pathway. Motile cilia generate a clockwise fluid flow, which leads to the asymmetric expression of *Nodal (Spaw*) and *Lefty* genes, thus establishing early body laterality.

The first cell fate determination during mouse embryo development occurs at the pre-implantation stage of the blastocyst, during which the inner cell mass either becomes the epiblast or the primitive endoderm, a predominantly extraembryonic tissue important for yolk sac formation to supply nutrients to the developing embryo and for embryo patterning [33,34] (Figure 4A). While the epiblast expresses high levels of the pluripotency factor Nanog, only low basal levels of ERK activity with sporadic pulses are detected. By contrast, the primitive endoderm expresses high levels of its markers GATA4 and GATA6 and has high levels of ERK activity [35,36]. These distinct ERK-activity patterns are regulated by FGF4-RAS signaling, where FGF4 is secreted by the epiblast to sustain the adjacent primitive endoderm [34,35]. After this initial differentiation step, epiblast cells differentiate into the three germ layers—endoderm, ectoderm, and mesoderm—during gastrulation.

### RAS signaling needs to be suppressed to maintain ESC and iPSC pluripotency

In vitro cultures of cells derived from the first embryonal layers are highly significant to understand molecular processes and explore biomedical applications of such pluripotent cells. Embryonic stem cells (ESC) are derived from the epiblast of mouse pre-implantation blastocysts from which the actual embryo derives (Figure 4A). In line with their origin, ESC have the potential to differentiate into all tissues of the adult, i.e. they are pluripotent regarding the development into the three embryonal germ layers. Furthermore, as common to all stem cells, they can self-renew, i.e., be propagated without differentiation [37].

The gene regulatory network of these cells is key to retaining pluripotency and includes transcription factors Nanog and the core pluripotency factors Oct4 and Sox2 [37]. In line with the low level of FGF4-RAS-ERK signaling determining the epiblast identity, from which ESC are derived, genetic elimination of *HRAS, NRAS,* and *KRAS* in mouse ESC prevents their differentiation but also reduces their growth [38].

To maintain ESC pluripotency *ex vivo*, the medium commonly referred to as ‘2iL’ was developed, which contains a Wnt pathway inhibitor blocking GSK3, the highly potent MEK1/2 inhibitor PD0325901 (‘2i’), and the cytokine LIF (‘L’) [39,40]. This is needed because autocrine FGF4 secretion triggers ESC to transition from the naïve to the primed pluripotency state, where the latter is already more restricted in its developmental potential [41]. Removal of the 2i enables transition of naïve ESC to the formative state, which is intermediate between the naïve and primed states and can respond to germline and lineage induction. During this process, RAS-ERK signaling removes Nanog expression while at the same time ensuring continued Oct4 expression, both of which are needed to up-regulate formative transcription factors [42]. Moreover, an ERK-independent effect of MAPK inhibition for pluripotency maintenance has been hypothesized, as complete ERK knockout causes a dysregulation of pluripotency genes and a self-renewal impairment in ESC [43]. Most likely, ESC require a minimal ERK level to correctly function, while a stronger activation leads ESC to differentiate [44] (Figure 4A).

One of the major biomedical research advancements in the past 30 years was the development of induced pluripotent stem cells (iPSCs), which allow the reprogramming of adult human tissue-derived cells to an embryonic-like pluripotent state [45]. In this process, somatic cells are reprogrammed by genetic (transduction with Yamanaka or OSKM factors Oct4, Sox2, Klf4, and Myc) or chemical compounds to become similar in their developmental potential to that of mouse ESC [46]. These cells can therefore serve as a source for tissue replacement and to provide starting material, e.g., for the generation of organoid models of genetic diseases [46]. In analogy to the maintenance of the naïve ESC state, the 2iL cocktail supports the generation of fully reprogrammed mouse iPSC consistent with a need to inhibit RAS signaling for iPSC establishment [47].

In conclusion, the FGF4-RAS-ERK signaling axis is critical already during early development when the first cell fates are determined. In vitro, this pathway needs to be suppressed to preserve the naïve pluripotency state of ESC and iPSC.

### RAS signaling is implicated in the regulation of key events during gastrulation

Gastrulation describes the period during embryogenesis from germ layer specification to establishment of the rudimentary body plan, notably the definition of the anteroposterior and dorsoventral axes [48]. During this process, cells do not only divide and become specialized but also reshape, migrate, and rearrange. Loss of FGFR1 signaling and thus RAS activity is embryonically lethal in mice at or before gastrulation [49,50].

Germ layer induction depends on subtleties of RAS activity during the stem cell cycles. We will therefore briefly recall the basics of cell cycle regulation. RAS-MAPK signaling is required twice during the G1 phase of the cell cycle. First, in early G1, the ERK activation by the RAS-MAPK pathway leads to cyclin D production. The subsequent assembly of the cyclin D-CDK4/6 complex is required for the phosphorylation of the retinoblastoma tumor suppressor (Rb) protein and the release of the E2F complex at the G1/S transition, allowing transcription of important cell cycle genes. In a second wave, in late G1, RAS signaling also induces the E2F-dependent expression of cyclin E, thus activating the cyclin E-CDK2 complex, which is required for the G1-S progression of the cell cycle. MAPK activity also drives the G1 phase forward with the initiation of CDK inhibitory protein degradation, given that e.g. p27^Kip1^ CDK inhibitor is high in the G0 phase [51,52]. However, very high RAS expression increases p38-MAPK signaling to induce p16^INK4A^, a CDK inhibitor that triggers senescence [53].

In pluripotent stem cells, unusually short G1 phases have been associated with their ability to proliferate rapidly and preserve stemness, as the time to integrate differentiation signals during G1 is reduced [54]. Sophisticated optogenetic experiments have demonstrated that exit from pluripotency depends on the accumulated ERK signaling, not duration, amplitude, or shape [55]. The tendency of the G1 phase-enriched ESC to differentiate was highest in cells with low levels of Rb phosphorylation [56].

In line with these data, the developmental trajectory of human ESC H9 cells is dependent on their position in the G1 phase. Early in the G1 phase, transcription factors Smad2 and Smad3 are nuclear, where they bind and activate endodermal genes [57]. Smad transcription factor activity is typically regulated downstream of morphogens of the TGFβ superfamily, including TGFβ, bone morphogenic proteins, growth and differentiation factors, activins, or Nodal [58]. Later in the G1 phase, high cyclin D levels increase the CDK4/6 activity, which increases phosphorylation of Smad2 and Smad3, leading to their exclusion from the nucleus, consequently preventing endoderm but permitting neuroectoderm differentiation [57].

In line with these data, dual Smad signaling inhibition blocks the formation of other germ layers and thus promotes neuroectoderm formation, i.e., the induction of CNS neural precursors, which served as a basis for a protocol for the accelerated derivation of functional cortical neurons from human iPSC [59]. Subsequently, cocktails of small molecules and growth factors allow for the relatively quick differentiation into neurons, while the derivation of oligodendrocytes is more time intensive [46].

### Embryonal patterning during somitogenesis is impacted by RAS activity

Gastrulation results in the formation of the primitive streak at the posterior side of the developing embryo. The anterior part of the primitive streak becomes the left–right organizer, an anatomical structure that exists only transiently during embryogenesis. Vertebrates display a pronounced left–right asymmetry both in their anatomy and in the positioning of their cardiovascular and gastrointestinal systems. During the development of most studied vertebrates, this is realized by left–right symmetry breaking in the so-called left–right organizer [60].

In most, but not all vertebrates, asymmetry is generated by the asymmetric expression, e.g., of diffusible TGFβ-superfamily members Nodal and Lefty. This is caused by the co-ordinated clockwise fluid flow (when viewed from the ventral side) that is propelled by motile ~6 μm long cilia on central cells of the organizer, while peripheral cells present immotile cilia in mouse [61]. It is thought that the generated leftward flow transports biochemical or mechanical signals that then manifest in asymmetric gene expression.

The left–right organizer of zebrafish (*Danio rerio*) is the Kupffer’s vesicle (KV), which is essentially a fluid-filled sac formed by ciliated cells (Figure 4B). FGF signaling and thus by inference RAS signaling appears to be important for mitotic events in the anterior part and early during the formation of KV [62]. Furthermore, the CXCR4 receptor is expressed in the KV primordium and induces, via the RAS pathway, the expression of cyclin D1 and thus the G1-S transition. This drives the proliferation of forerunner cells of the KV. Moreover, cyclin D1-CDK4/6 phosphorylate Foxj1a in these cells, preventing its ubiquitin-independent proteasomal degradation. Foxj1a is considered a master transcriptional regulator of motile cilia, which explains how CXCR4 promotes KV formation [63].

Later during development, somites arise as segments in the transient paraxial mesodermal structures along the neural tube. Somites subsequently give rise to skeletal muscle, axial skeleton, and dermis. Somitogenesis progresses along this principal body axis in anterior–posterior direction, such that new somites sequentially form anteriorly of the growing unsegmented caudal tissue. It is not yet completely understood how the emerging repetitive pattern of somites is formed, and various models exist to explain the segmented body plan. However, essentially all models involve FGF-RAS-ERK signaling, which further cross-talks with other developmental pathways. An FGF gradient (non-cell autonomously) here encodes the information on where to create somite boundaries, which are thus determined by a fixed, high ERK activity gradient between neighboring cells. In addition to this positional information, ERK activity also oscillates under the control of developmental transcription factors (cell-autonomously) of the Notch pathway (i.e. Hes/Her family) to provide temporal information of when to form a boundary. More recent models partially combine these two ideas of oscillation and ERK-activity gradient [64].

**Figure 5:**
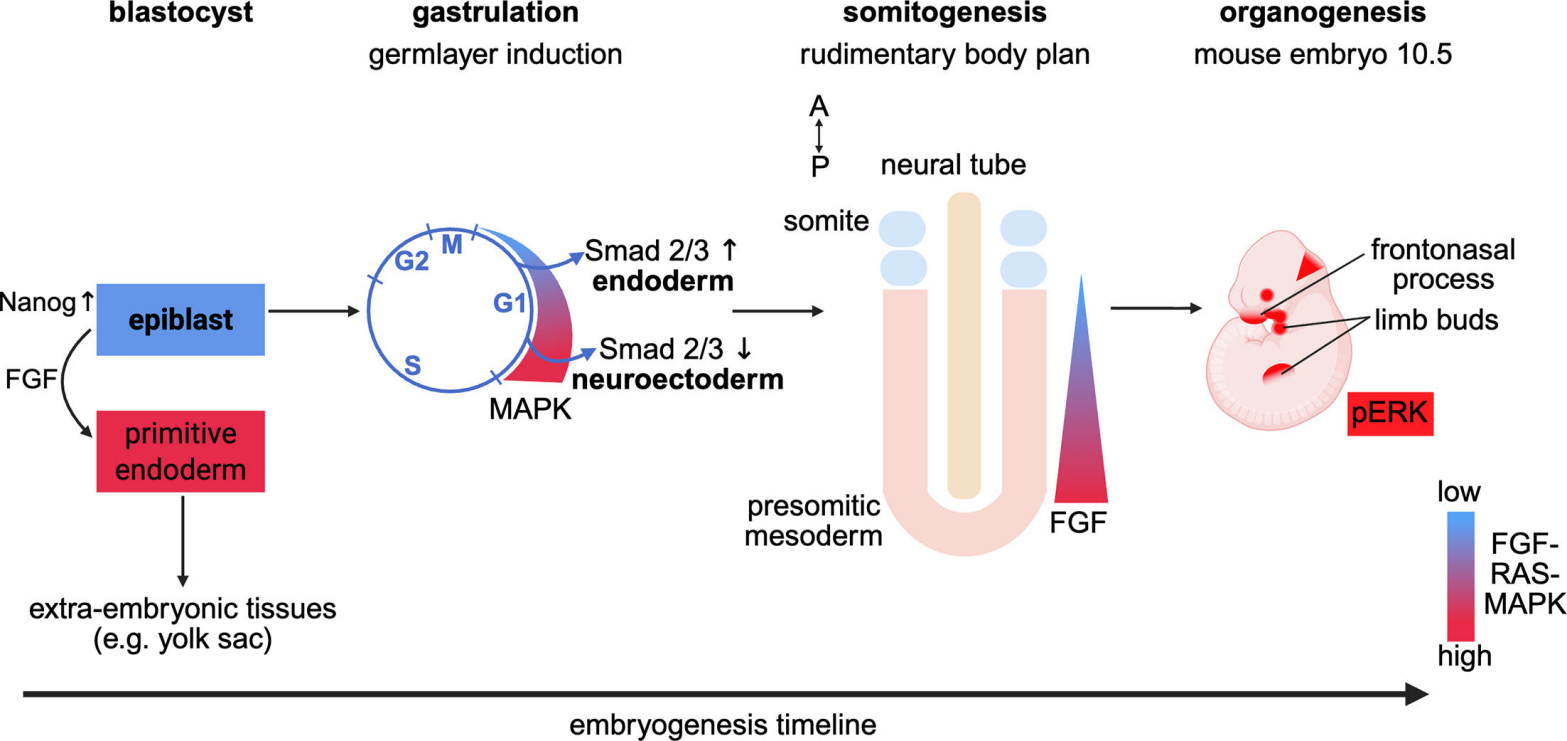
FGF-RAS-MAPK pathway activity during embryogenesis. High- and low-pathway activity is observed at various stages (top) throughout development and spatio-temporally highly co-ordinated. Therefore, any correction of its aberrant function would require the same precision. See text for more details about the impact of RAS signaling at each of these stages.

The importance of RAS signaling during these critical embryonal stages can probably be generalized. Immunohistochemical visualization of active ERK1/2 can help to infer RAS-MAPK pathway activity. Applying this method during mouse E5-10.5 stages revealed RAS-MAPK activity approximately from the implanted blastocyst to about midway through embryonal development, where the somites are formed and neural, limb, and heart anlagen are in establishment [65]. This helped to identify distinct regions of ERK activity that were dynamically changing over time, such as in the limb buds, parts of the brain, foregut, and liver. Again, many, but not all of these regions overlapped with regions of high FGF expression (Figure 5).

### RAS isoforms appear to have overlapping yet distinct roles during development

As illustrated above by the last examples, direct observation of RAS activation itself was rarely accomplished. Instead, the distribution of active, phosphorylated ERK serves as a proxy. Alternatively, the developmental expression pattern of RAS can give an idea of where RAS could be active during development. However, few examples exist where the expression of individual RAS isoforms during development was investigated.

When comparing the transcripts of the cancer-associated RAS isoforms from embryonal development to the adult in major tissues, a clear dominance of KRAS4B expression amounting to 60–99% of all RAS transcripts was observed [66]. This strongly suggests a pivotal function of KRAS4B during the formation of all tissues. Expression studies of an endogenously tagged *KRAS* gene support a higher expression during embryonal development, which is somewhat down-regulated in the adult [67].

It is furthermore evident that the expression of the different RAS isoforms varies during development in a tissue-specific manner [66]. Hence, while RAS proteins are ubiquitously expressed, their expression pattern is also consistent with distinct functions during recurrent processes of development and potentially specific functions in different tissues. These data are corroborated by protein expression data from various tissues in adult mice, where KRAS4A/4B was typically more abundant than the other two RAS isoforms combined, followed in abundance by HRAS and much less NRAS [19]. Tissues with relatively high expression of HRAS were the cerebral cortex and skeletal muscle.

**Figure 6:**
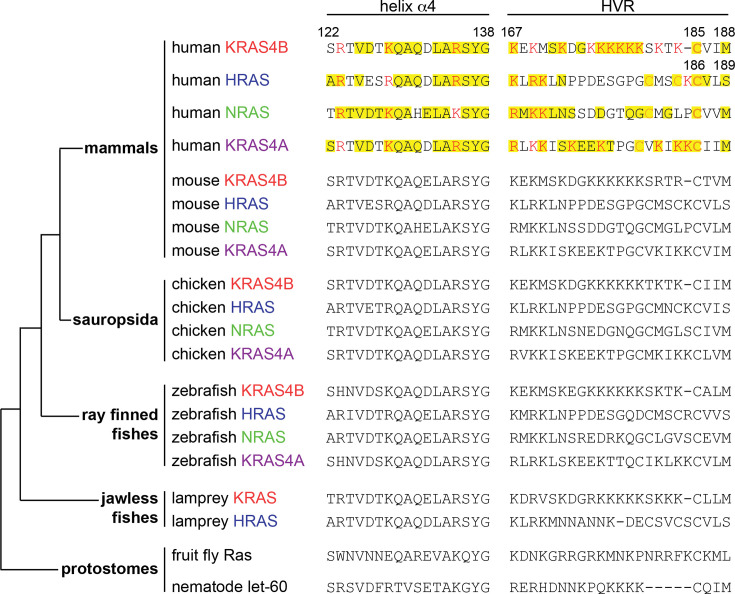
Phylogenetic tree of RAS isoform evolution. Simplified phylogenetic tree based on that from Garcia-Espana et al. [68]. The alignment of the most divergent sequence stretches within the RAS sequence, helix ⍺4, and the C-terminal hypervariable region (HVR) [69] was generated using Clustal Omega Multiple Sequence Alignment (1.2.4) [70]. Basic residues are printed in red, while lipid-modified cysteines are in orange. Conserved residues are highlighted in yellow in the human sequences.

The importance of *KRAS* during development is supported by the fact that the gene for KRAS4B is the most ancient. It was followed by the *HRAS* gene, which emerged in jawless fish, and only then did genes for the other RAS proteins arise [1] (Figure 6). Another important argument for the critical role of KRAS4B comes from mouse knockout studies, where only the knockout of *KRAS* is embryonically lethal [71,72], and the splice variant KRAS4A is dispensable [73]. The same is true for knockouts of *NRAS* and *HRAS,* which are regarded as dispensable for mouse embryonal growth and development [74,75]. However, homozygous deletion of *NRAS* and heterozygous deletion of *KRAS* also lead to embryonal death, suggesting that compensation by other RAS isoforms requires at least a certain gene dose, if not a sufficient expression of *KRAS* specifically [72]. An interesting observation was made in mice where essentially *HRAS* was expressed from the *KRAS* gene locus. These mice were viable but had dilated cardiomyopathy, suggesting that *HRAS* cannot fully replace *KRAS*, but that in this context, *KRAS* may be dispensable [76]. Given that germline point mutations in *RAS* genes can lead to significant developmental malformations in syndromes, referred to as RASopathies (see below), it is plausible to assume that *NRAS* and *HRAS* knockout mice are not without phenotypic defects. In fact, double-knockouts of *HRAS* and *NRAS* display a RASopathy-like phenotype [77], which contrasts to the assessment of the same double-knockout 25 years ago [75], i.e. at a time when many other *RAS* knockout studies were conducted and evaluated.

## Aberrant RAS activity during development causes RASopathies and can promote cancer in the adult

Genetic diseases in humans allow a similar insight into the activity of RAS during development as experiments with transgenic animals. Vice versa, animal models of these diseases give detailed insight into the perturbed molecular and cellular processes.

The RASopathies are a collection of individually rare but collectively common rare diseases where RAS pathway genes are heterozygously mutated in the germline [78,79]. In contrast with RAS pathway-driven cancers, a single disease-causing allele usually up-modulates RAS-MAPK activity and drives developmental abnormalities that are already observable during gastrulation [14,80]. The core symptoms are shared across all RASopathies and manifest progressively. They can significantly affect the quality of life of affected children (musculoskeletal anomalies, gastrointestinal and cutaneous issues, neurocognitive and behavioral deficits) and can be life-threatening (pulmonary insufficiency, rare cancers, seizures, and hypertrophic cardiomyopathy). Severe symptoms are particularly evident in the more common RASopathies, neurofibromatosis type 1, and Noonan syndrome, and in the rare cardio-facio-cutaneous syndrome [79].

Neurofibromatosis type 1 is caused by heterozygous loss-of-function mutations in the gene encoding NF1, the most critical RAS-GAP by disease association (Figure 1A), while both *KRAS* and *NRAS* mutations were described to cause Noonan syndrome [78]. An attenuated phenotype from that in neurofibromatosis type 1 is seen in the very rare Legius syndrome, where the gene of the NF1-recruiting SPRED1 is mutated [81]. By contrast, loss of *SPRED2* function leads to Noonan syndrome [82]. Although *HRAS* mutations are less common, the *HRAS-G12S* mutation is the predominant one in the very rare Costello syndrome. The most frequently mutated RASopathy gene is *PTPN11*, which encodes the tyrosine phosphatase SHP2 that is thought to act upstream of RAS. Interestingly, both gain- and loss-of-function mutations of this gene were associated with RASopathies [83,84]. This biochemical duality is also seen in RASopathy-associated KRAS4B mutants, where gain- and loss-of-function features within the same protein appear to manifest at different stages of cell differentiation [21]. Several observations extend the commonly accepted model that RASopathies are driven by mild RAS-MAPK pathway overactivation, as compared with what is observed in cancer. We surmise that RAS-associated cell differentiation and thus organismal development are fundamentally dysregulated by the subtle pathway activations. In the following, we will discuss findings from human patients and of vertebrate RASopathy models, which support that organismal development is perturbed early, and tissue differentiation, such as of muscle and brain, is severely disrupted.

The zebrafish is a classical vertebrate model organism in developmental biology, which has provided significant insight into RASopathies [85]. Both gain- and loss-of-function mutations in zebrafish *Shp2* cause heart defects and randomized left–right asymmetry [86]. Both ciliogenesis and cilia function in KV were defective in mutant animals, confirming severe observable developmental defects as early as gastrulation [80]. Consistent with the mild RAS-MAPK pathway activation by RASopathy mutations, inhibition of MEK during the development of mutant embryos could rescue these defects almost to control levels. Intriguingly, ciliation defects in KV are also widely observed in other RASopathy models, suggesting a potential implication of the RAS pathway in ciliogenesis [85].

In human RASopathy patients, the accumulated defects during development manifest in the above outlined spectrum of symptoms. As compared with cardiac and neurological abnormalities, musculoskeletal malformations are somewhat neglected symptoms in RASopathies, even though associated feeding difficulties in infants increase morbidity [87]. However, given that we here provide significant mechanistic background on muscle development, we would like to emphasize that in line with the described role of RAS-MAPK signaling in this process, also muscle weakness is a primary phenotype of RASopathies, which is reflected in several parameters, such as reduced muscle size and decreased lower body and hand-grip strength [87,88]. Based on this, it is, for instance, conceivable to employ handgrip strength as a biomarker for general therapy responsiveness in these diseases, as this parameter is quite easily and non-invasively assessed.

These findings are reflected in mouse models. Homozygous loss of NF1 in mice results in early embryonic lethality with embryos exhibiting abnormal cardiac and neural crest-derived tissue development, as well as a delay in renal, hepatic, and skeletal muscle development [89]. Similarly, knockout of NF1 only during embryonal limb development (NF1_Prx1_^-/-^) reduces muscle mass and strength. During embryogenesis, this is characterized by a reduction in markers of differentiating muscle cells [90]. In the mouse model for Costello Syndrome, the *HRAS-G12V* variant is expressed in the germline [91]. In the embryo, the expression of the muscle stem cell marker Pax7 is increased in certain muscles, while markers for terminal muscle differentiation are reduced after birth but recover with MEK inhibition [92]. This overall establishes a perturbed muscle differentiation in RASopathies and supports a positive role of RAS signaling in stem cell proliferation, but a need for its inhibition during terminal differentiation [87].

*In vitro*, transformation of a muscle cell line that contains both stem cells and committed myoblasts with RASopathy variants of KRAS4B confirmed that both the stem cell population and terminal differentiation can be negatively impacted [21]. Deficits in sustaining the stem cell population may be surprising, given that mutants are considered to mildly overactivate the MAPK pathway. However, closer inspection of the biochemical properties of studied mutants revealed that they share both gain- and loss-of-function features [21]. This is illustrated by the KRAS4B-G60R mutant, which shows decreased effector binding (loss-of-function) and is also compromised for NF1-GAP-stimulated hydrolysis (gain-of-function) like NF1-GAP-resistant oncogenic RAS mutants. This G60R mutant was associated with a loss of muscle stem cells but also a block of terminal muscle cell differentiation [21]. Given that all oncogenic RAS variants blocked terminal differentiation in this study, it can be inferred that the G60R loss-of-function characteristic caused the loss of the stem cell pool, while the gain-of-function feature (NF1-GAP-resistance) is associated with the block of terminal differentiation.

Cell differentiation in the developing brain is highly complex with multiple rounds of spatio-temporally co-ordinated cell specifications [31]. Disentangling the role of the RAS pathway in this process or during plastic adaptations in the adult will therefore be an ultimate future challenge. The simplified differentiation scheme where neural stem cells give first rise to both neuronal and glial progenitors, with the former further specializing into different neurons, while the latter giving rise to both astrocytes and oligodendrocytes [93], may, however, serve as a scaffold to organize the following observations.

Expression of oncogenic *KRAS-G12D*, but not *HRAS-G12V* or *NRAS-G12D*, increased the number of astrocytes, but not oligodendrocytes or neurons in the brain of young mice, while also the neural stem cell pool itself expanded in the same manner [94]. Earlier data examined the impact of Costello syndrome-associated *HRAS-G12V* and *HRAS-G12S* on cortical neuron development *in vitro* and after electroporation *in utero*. Here, neurogenesis was inhibited, while gliogenesis with increased proliferation of astrocytes was promoted [95]. Faster glial differentiation and proliferation were likewise observed in patient-derived iPSCs carrying an *HRAS-G12S* mutation [96].

Given the NF1-GAP resistance of oncogenic RAS mutants, their phenotype should partly be emulated by loss of NF1. Indeed, conditional *NF1* deletion in radial glia, which give rise to all neuronal and glial lineages during early brain development, shows increased gliogenesis at the expense of neurogenesis. This results in an enlarged subventricular zone with increased glial differentiation [97]. Given that some of the resulting structural defects only manifested in the neonate, administration of a MEK-inhibitor during this period could significantly rescue the phenotypic abnormalities [97]. In line with the above observations, the Noonan syndrome mouse model expressing RAF1-L613V displayed an increase in astrocytes and oligodendrocyte progenitors in the cortex [98]. Also, the cardio-facio-cutaneous syndrome mouse model expressing Mek1-Y130C and its wildtype counterpart increased cortical and hippocampal densities of both astrocytes and oligodendrocytes and their precursors [99]. It thus appears that RASopathy mutations increase gliogenesis eventually at the expense of neurogenesis.

In conclusion, by acting throughout development, RASopathy disease variants perturb organismal development from the bottom up. It is not plausible that accumulated developmental defects can be repaired postnatally. An important exception may be brain development, which both in mice and humans largely continues postnatally. Likewise, tissues that undergo significant development (growth) and turnover postnatally or in the adult, such as the musculoskeletal system, may benefit from therapeutic interventions. A major issue for a timely intervention is that of an early diagnosis of a RASopathy [100]. It will, however, be a major challenge to find solutions for the fact that some tissue development processes need a down-modulation of the pathway (e.g. muscle terminal differentiation), while others require its up-regulation (e.g. muscle stem/progenitor cell proliferation) (Figure 3). The success of MAPK-pathway inhibitors to achieve partial rescue can be explained by the fact that proper terminal differentiation of most tissues may require MAPK-pathway down-regulation, as opposed to the mild up-regulation seen with RASopathy mutants.

Deep insight into the molecular and cellular states that bear the genetic defect may potentially allow reprogramming cells pharmacologically in situ from an aberrant to a normal differentiation trajectory.

### From RASopathies to cancer

RASopathies can furthermore predispose to certain cancers, such as muscle tissue-associated rhabdomyosarcoma. It is the most common childhood soft-tissue sarcoma with only 30% survival of patients suffering from the metastatic disease [101]. Rhabdomyosarcoma is driven by either *RAS* mutations or RAS pathway activation, which blocks myogenic differentiation [102]. Inhibition of MEK slows the growth of rhabdomyosarcoma xenografts and in combination with RAF inhibitors, such as Regorafenib or Dabrafenib, promotes myogenic differentiation and blocks tumor growth [102,103].

Muscle cancer studies in zebrafish suggest that only transformation of the stem cell or early progenitor cell compartment with KRAS-G12D resulted in less differentiated muscle tumors associated with poorer survival, as compared with expression in differentiated muscle cells [104,105]. The tumor-derived subpopulation that is high in expression of Myf5, a myogenic transcription factor that is induced in activated tissue-resident stem cells and high in proliferating myoblasts, was enriched in cells with tumor-propagating activity [105]. Mouse experiments support the origin of rhabdomyosarcoma in cells that originate from the tissue-resident muscle stem cells and not from non-myogenic, i.e. fibrogenic and adipogenic progenitors [106].

Perturbed differentiation due to RAS-pathway gene mutations is also observed in brain tumors. The most aggressive brain tumor that accounts for 50% of all malignant brain tumors is glioblastoma, and the five-year survival rate for all malignant brain tumors is only 36% in the USA [107]. While still somewhat contentious for many cancer types, for brain tumors, it is well accepted that they mostly emerge from a neural progenitor with stem-like properties [108]. Brain tumors are derived from stem and progenitor cells of the neural crest cell type (neuroblastoma), neural stem or progenitor cell (medulloblastoma) lineages, while malignant glioma can be derived from neural stem cells, glial progenitors, or astrocytes. While RAS itself is not typically mutated, the RAS pathway is genetically altered in glioblastoma, astrocytoma, and high- and low-grade glioma, with NF1 loss being quite common in up to 17% of glioblastoma [109]. Based on the above-mentioned phenotypes that are associated with a loss of NF1, this alteration typically promotes a proliferative glial fate, while fewer neurons are formed during differentiation.

Oncogenic RAS perturbs differentiation also in other tissues. In genetically engineered mice, expression of oncogenic *KRAS-G12D* in the colonic epithelium, but not of *NRAS-G12D*, drives hyperplasia in the colon by increasing proliferative progenitor cell numbers. When combined with loss of Apc (adenomatosis polyposis coli tumor suppressor), terminal differentiation was impaired, leading to an accumulation of stem-like cells [110]. In line with this, oncogenic *KRAS* induces a stem cell-like gene expression program in human colon cancer by reactivating an ESC-like transcriptional signature and not just proliferation [111]. KRAS4B, but not HRAS, is also the target of an experimental cancer stem cell drug salinomycin. Salinomycin is an antibiotic that was identified by the Weinberg and Lander groups as having activity against cancer cells expressing breast cancer stem cell markers [112]. It indirectly targets KRAS4B by disrupting its phosphatidylserine-dependent nanoscale membrane organization [113]. It was shown to be particularly active in cancer cells having a KRAS4B-associated and ESC-like gene expression signature [113]. This signature comprises only 9 genes, including those for caveolin-1, cavin-1, and others that are known to affect KRAS4B membrane organization. This was in line with the mechanism of action of salinomycin against KRAS4B. The signature is present in 8% of TCGA (The Cancer Genome Atlas)-tumor samples while also being associated with a significantly higher mortality, suggesting that embryonic KRAS4B programs could be a focus of future anti-cancer stem cell drug investigations.

**Figure 7:**
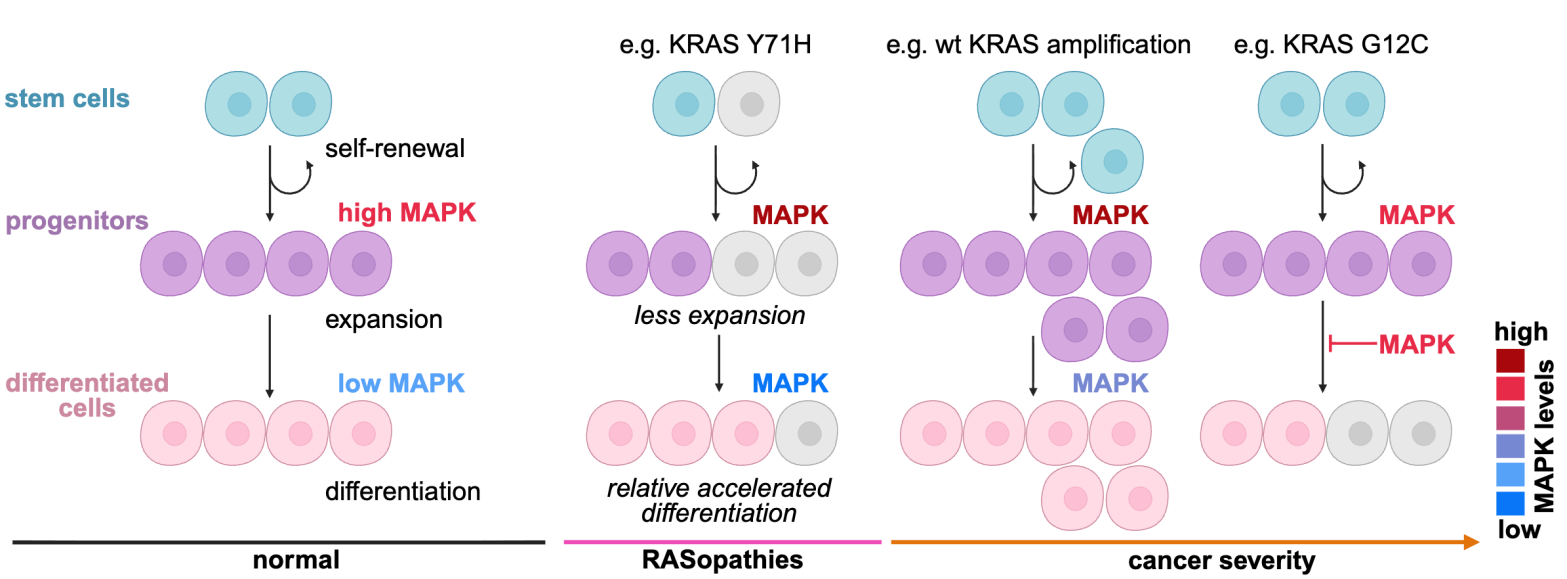
Proposed simplified model of the effect of RAS activity modulation on cell differentiation in health and disease. While normal stem cell and progenitor proliferation requires high MAPK levels, the pathway must be transiently down-regulated to permit terminal differentiation. Subsequent up-regulation during tissue maturation is implied. Some RASopathy mutants, such as KRAS-Y71H, appear to have mild gain- and loss-of-function biochemical features, which may each manifest predominantly at different stages of differentiation. KRAS-Y71H, for instance, was associated with reduced stem cell numbers as compared to normal (gray cells, mean not present vs. normal) yet produced an almost normal level of differentiated cells. In cancer, KRAS amplification or mutation is expected to have different effects on cell differentiation, as the latter is insensitive to the NF1-dependent licensing of terminal differentiation, which halts cells in the immature and proliferative progenitor state. We hypothesize that pharmacological interventions are mostly observed on the many cells of the tumor mass (pink and purple) but not potentially founding cells of the stem cell compartment (turquoise). Cancer cell lines may be somewhat representative of the cells in the tumor mass. However, to correct aberrant activities at the origin, in the founder cells at the stem cell/progenitor level, different rationales than those currently in place may have to be applied, which may even require restricted MAPK-pathway activation.

In conclusion, we suggest that RAS proteins are probably among the most important proteins during development, where already subtle malfunctions can lead to RASopathies. We propose that, in RASopathies, cell differentiation is broadly perturbed throughout development, where loss-of-function features (with lower RAS effector engagement) manifest in the stem/progenitor cell compartment, whereas gain-of-function mutants specifically with a loss of NF1-GAP sensitivity block terminal differentiation, such as observed with oncogenic RAS mutants (Figure 7).

## The distinct roles of negative regulators of the RAS-MAPK pathway: SPRY consolidates quiescence while NF1 promotes terminal differentiation

We just learned that any dysregulation of normal RAS activity, notably its compromised inactivation during terminal differentiation, can lead to serious diseases during development or repair in the adult. In this last chapter, we will therefore review cardinal molecular processes that negatively regulate RAS during development.

Sprouty (SPRY) proteins have been identified genetically in Drosophila as negative regulators of MAPK signaling [114]. After FGF-induced plasma membrane translocation [115], they are generally believed to block RAS activity either upstream [116] or downstream of RAS [117,118]. High SPRY1 expression has been associated with quiescence of muscle stem cells, suggesting it consolidates this state by down-regulating RAS-MAPK signaling [119]. This cell cycle-arrested state is distinct from that of post-mitotic differentiated cells, as quiescence is reversible. Upon injury-induced stem cell activation, SPRY1 is down-regulated and again elevated as cells re-enter quiescence. Importantly, this process is perturbed during aging, where elevated FGF2 activity disrupts quiescence of muscle stem cells, which then lose their self-renewal capacity [120]. In addition, epigenetic changes appear to contribute to SPRY1 suppression [121].

As mentioned before, the most prominent negative regulator of RAS is the GAP NF1, which cannot act on oncogenic RAS mutants. It was furthermore suggested that any higher affinity of RAS mutants to NF1 may enable partial sequestration of NF1 by such a mutant and thus relatively activate wildtype RAS [122]. In vitro data showing that terminal differentiation of muscle cells is blocked by any oncogenic RAS mutant supports a critical involvement of NF1 in this process [21]. In line with this, treatment with a MEK inhibitor during development could not rescue the muscle deficits in newborn mice where NF1 is deleted early during limb development (NF1_Prx1_^-/-^), while the same treatment in a related model where NF1 was deleted in committed progenitors just before terminal differentiation (NF1_MyoD_^-/-^) was successful [123].

What is the molecular mechanism that allows NF1 to be engaged in the inactivation of normal, non-mutant RAS? Some 25-year-old data from the C2C12 cell model for *in vitro* muscle differentiation suggest that SPRED1 and NF1 are induced once terminal differentiation is triggered [124,125]. This would allow SPRED1 to recruit NF1 to inactivate RAS.

The interaction of these two proteins is, however, further regulated. The N-terminal EVH1 domain of SPRED1 mediates the high-affinity interaction with the non-catalytic GAP-related domain of NF1 [11,126]. However, phosphorylation of S105 in SPRED1 by CDK1 disrupts NF1 binding [11]. Given that the RASopathy-associated mutation T102R is in close proximity to S105 and showed an even stronger phenotype in a cell competition assay than the phosphomimetic mutant SPRED1-S105D, it is plausible to assume that T102 in the EVH-1 (Ena/VASP Homology 1) domain of SPRED1 is also contributing to the above regulation [11,81].

The CDK1 dependence may imply that the last cell cycle of committed progenitors enables the terminal differentiation to ensue, after all RAS has been inactivated by NF1. Alternatively, cells undergo a transition utilizing an alternative cell cycle, such as recently described for the differentiation of multiciliated tracheal epithelial cells [127]. In that model, differentiating cells utilized the cell cycle machinery from G0 through S and G2/M up until the end of the G1 phase, while DNA replication and S phase-like gene expression were blocked via induction of the E2F7 transcriptional regulator. In any case, the interaction between NF1 and SPRED proteins must be enabled by the activity of a currently unknown phosphatase that counteracts the CDK1 activity.

In summary, data from muscle development suggest that SPRY and SPRED/NF1 have roles at the opposite ends of the differentiation trajectory, i.e., in quiescent stem cells and differentiating cells, respectively. The predominant view on these negative regulators acting on RAS in cancer cells concurrently may therefore be simplified, as it does not take the natural developmental or tissue context into account.

## Conclusions and perspectives

Already some of the earliest model organism data have shown that RAS functions by regulating both cell proliferation and cell differentiation. RAS signaling is important from the first steps in embryonal development through to the maintenance of adult tissues. We propose that the set of ~20 different human RAS family proteins are utilized during the specialization of the various tissues of our body. The evolutionary most ancient KRAS4B may be particularly relevant to maintaining stemness, while other RAS isoforms adopt their distinct yet overlapping activities during other stages of cell differentiation and tissue specification. The impact of RAS activation on the fate of a cell may therefore depend on the isoform, while we typically only have data downstream of RAS itself, such as ERK activity.

On the one hand, MAPK activity needs to be suppressed to maintain pluripotent stem cells and to preserve adult stem cell quiescence, while on the other hand, terminal differentiation seems to require NF1-GAP-mediated RAS inactivation, as suggested by muscle differentiation data. Given this deep implication in the most fundamental processes of metazoan life, it is not surprising that even the most subtle biochemical aberrations in RAS-MAPK functioning, such as those observed in RASopathies, have a detrimental impact on organismal development. Hence, defects that are established in the newborn are unlikely to be rescued by postnatal therapies. However, given that many tissues continue to mature during childhood, their erroneous developmental trajectories due to RAS pathway mutations should be amenable to pharmacological correction. The task at hand for both cancer and RASopathy therapy development is nothing less than to reprogram the aberrant differentiation of affected tissues exactly back to normal. To accomplish this, we will need to understand in detail how the different RAS isoforms affect cell differentiation and tissue specialization. Yet, encouragingly, animal experiments have demonstrated that MAPK pathway inhibitors can indeed afford a certain level of correction in both RAS-driven diseases. We postulate that those pathologies that are progressive, i.e., where significant aberrant differentiation occurs postnatally during infancy, should have a greater potential for pharmacological rescue.

We furthermore propose the impact of RAS-MAPK activity on cell differentiation as the common denominator of aberrant RAS functioning in RASopathies and cancer. This new perspective should not only shape our expectations regarding the possibilities and limitations of treating these diseases. It furthermore should tell us that we need more cellular differentiation models to understand molecular details not only of RASopathies but also of cancer much better. Such cellular models will be critical to developing novel differentiation-correcting drugs. However, current trends in cellular cancer models focus on the complexities of the tumor tissue, which hosts a dazzling diversity of cell types in the tumor microenvironment. Clearly, this approach is important to deal with tumors as they are typically diagnosed, as the ‘wounds that do not heal’ [128]. Yet, we may wish to remember that in most cases, malignant tumors do not develop, as the body is able to repair and ‘heal’ itself by utilizing normal cell differentiation paths. It is conceivable that these repair efforts could be pharmacologically supported to avert cancer and related diseases at old age.

Finally, we expect that single-cell RNA sequencing has the potential to open the door to unravel not only the cellular complexities of the tumor ‘wound’ but in particular to help us understand how normal tissue repair is functioning [129,130]. This technology ushers in a new age not only of developmental biology, where multiple successive cell differentiation hierarchies can be interrogated. We speculate that single-cell transcriptomics data may reveal signatures of recurrent RAS pathway usage as cells become increasingly specialized. This is supported by the fact that all core proteins of the RAS pathway, in particular KRAS4B, NRAS, and HRAS, are evolutionarily conserved and are critical for organismal development (see RASopathy phenotypes) and are exploited in almost every cancer. We argue that, by having a representative model of such a core RAS differentiation module, we can employ it to understand and manipulate RAS signaling in its native molecular context, as compared to its function in highly heterogenous, dysfunctional cancer cells. It will be interesting to see if single-cell resolution techniques can help us to place RAS back on track to heal tissues during postnatal development and repair.

## Open Access

This article has been published open access under our Subscribe to Open programme, made possible through the support of our subscribing institutions, learn more here: https://portlandpress.com/pages/open_access_options_and_prices#conditional

## Abbreviations

CNS: central nervous system
ESC: embryonic stem cells
GAPs: GTPase-activating proteins
GEFs: guanine nucleotide exchange factors
KV: Kupffer’s vesicle
NF1: neurofibromin 1
SPRY: Sprouty
iPSCs: induced pluripotent stem cells
